# Teaching Occlusal Splints in the Digital Age: Comparing Student Experiences With Conventional and CAD/CAM Workflows

**DOI:** 10.1002/jdd.70045

**Published:** 2025-09-10

**Authors:** Marcelo José Palma‐Fernandes, Júlio Ruiz‐Marrara, Maria Fernanda de Campos‐Muller, Melissa Oliveira de Melchior, Inês Sansonetty Gonçalves Côrte‐Real, Jardel Francisco Mazzi‐Chaves, Laís Valencise Magri

**Affiliations:** ^1^ Faculdade De Medicina Dentária Da Universidade do Porto Porto Portugal; ^2^ Departamento De Prótese e Materiais Dentários, Faculdade de Odontologia de Ribeirão Preto Universidade De São Paulo São Paulo Brasil; ^3^ Departamento De Odontologia Restauradora, Faculdade de Odontologia de Ribeirão Preto Universidade De São Paulo São Paulo Brasil

**Keywords:** CAD/CAM, digital dentistry, occlusal splint, student perceptions

## Abstract

**Background:**

The teaching of occlusal splint therapy in dental education is evolving with the integration of digital workflows. Although digital tools offer operational advantages, conventional methods remain pedagogically relevant. Understanding students’ perceptions of both approaches is essential for guiding curriculum innovation.

**Aim:**

To evaluate undergraduate dental students’ perceptions of conventional and digital workflows in occlusal splint fabrication and analyze the perceived educational benefits and limitations of each method.

**Methods:**

This mixed‐methods study included 24 undergraduate dental students who fabricated occlusal splints using both workflows. Quantitative data were analyzed using descriptive statistics, Bland–Altman plots, and intraclass correlation coefficients (ICCs) to assess agreement on ease of fabrication, satisfaction with outcomes, and likelihood of recommendation. Qualitative responses underwent lexical analysis and were visualized using word clouds.

**Results:**

The digital workflow received higher scores for ease of fabrication (mean: 9.0 ± 0.9) versus the conventional method (mean: 4.0 ± 1.1), with a significant mean difference evident in the Bland–Altman plot. Satisfaction with results was comparable between workflows (digital: 9.0 ± 0.7; conventional: 8.6 ± 0.8; ICC = –0.416). Recommendation scores favored the digital workflow (digital: 9.6 ± 0.6; conventional: 6.0 ± 1.2; ICC = 0.62). Qualitative analysis associated digital workflows with “fast” and “efficient,” and conventional techniques with “learning” and “understanding.”

**Conclusion:**

Digital workflows enhance efficiency and are well‐received by students, while conventional methods support critical technical development. A blended instructional model is recommended to optimize clinical education in the digital era.

## 1 Introduction

The teaching of occlusal splint therapy is fundamental in undergraduate dental education, as it provides future clinicians with essential competencies to diagnose and manage functional disturbances such as temporomandibular disorders (TMDs), bruxism, and other occlusion‐related conditions. Despite the critical role in restorative and prosthetic dentistry, substantial variability exists in how these topics are taught across institutions. In many cases, theoretical instruction is not adequately reinforced by clinical application, which can compromise students' confidence and competence in real‐world practice.

In the United Kingdom and Ireland, for instance, although all dental schools include occlusion in their curricula, there is no standardized framework that defines core content, duration, or integration with clinical practice [1]. The situation is similar in Brazil, where studies have demonstrated inconsistencies in the curricular inclusion of TMDs and orofacial pain (OFP) [2]. Furthermore, even when TMD‐related content is present, it often lacks structured clinical training and is rarely aligned with the evolving paradigms of diagnosis and treatment [1, 3]. These gaps reflect a broader global trend in dental education, where foundational knowledge in occlusion and related therapeutic interventions, such as occlusal splint fabrication, is unevenly distributed, leading to educational deficits in clinical preparedness [2, 3].

Teaching occlusal splints specifically remains underexplored in the literature. While occlusal splints are recognized as an effective non‐invasive treatment for TMDs and sleep bruxism, their instruction at the undergraduate level is often limited to theoretical lectures or demonstrations without sufficient hands‐on experience. The lack of consistency in teaching methodology and divergent occlusal philosophies contributes to a fragmented understanding of splint therapy among students [1, 2]. This fragmentation can hinder students' ability to critically evaluate treatment indications and develop the necessary technical skills for clinical implementation [3].

Technological innovation offers an opportunity to bridge these gaps. The integration of digital workflows into dental education, including intraoral scanning, CAD/CAM design, and 3D printing, has transformed the way students engage with prosthetic and occlusal concepts [3]. These tools not only enhance procedural efficiency but also foster visualization and spatial reasoning skills critical for understanding complex anatomical and functional relationships [3]. In particular, temporomandibular joint (TMJ) anatomy, occlusion, and mandibular dynamics have been identified as priority areas for the incorporation of 3D learning resources and virtual simulation environments in dental training [3]. Such resources also align with students’ perceived learning needs, as they offer opportunities to interact with content in an immersive and flexible manner, facilitating deeper conceptual understanding [3].

Nonetheless, few studies have investigated how undergraduate students perceive the learning process when exposed to both conventional and digital workflows for occlusal splint fabrication. Understanding these perceptions is crucial for designing pedagogical strategies that align technological adoption with meaningful learning [2, 3]. Mixed‐method research that combines quantitative evaluation with qualitative analysis of student experiences can provide nuanced insights into how different teaching approaches affect skill acquisition, critical thinking, and clinical readiness. Moreover, identifying learners’ perspectives helps ensure that curricular innovations are grounded in authentic educational needs, rather than assumptions made by educators or technology developers [3].

This study aims to explore undergraduate students' perceptions of conventional and digital workflows in occlusal splint fabrication, identifying educational benefits and challenges of each. The goal is to inform curriculum development with evidence‐based recommendations for integrating innovative technologies into occlusal therapy training.

## 2 Methods

### 2.1 Study Design

This educational research employed a mixed‐methods, cross‐sectional design to explore dental students’ learning experiences and perceptions regarding two workflows for occlusal splint fabrication: digital and conventional. The primary pedagogical goal was not merely to compare clinical techniques but to promote reflective learning, critical appraisal skills, and technological adaptability within a competency‐based dental education framework. Ethical approval was obtained from the Research Ethics Committee of the School of Dentistry of Ribeirao Preto, University of Sao Paulo (FORP/USP) (CAAE protocol 83146624.8.0000.5419).

The educational activity was integrated into the Temporomandibular Disorders and Orofacial Pain course, a required discipline for seventh‐semester undergraduate dental students. It was designed based on experiential learning theory, emphasizing active engagement, hands‐on practice, and guided reflection as core strategies to strengthen clinical reasoning and procedural proficiency. Faculty members functioned as facilitators throughout the activity, offering formative feedback and promoting student autonomy during the fabrication process.

A mixed‐methods approach was adopted to allow for a comprehensive understanding of both measurable student preferences and qualitative insights into their learning processes. This triangulation of data sources contributed to a robust educational evaluation, grounded in student‐centered pedagogical principles.

### 2.2 Participants

A total of 24 undergraduate dental students participated in this study. All were enrolled in the seventh semester of the Dentistry program at the School of Dentistry of Ribeirao Preto, University of Sao Paulo (FORP/USP), and were attending the required course on Temporomandibular Disorders and Orofacial Pain, which includes hands‐on clinical training in occlusal splint therapy.

Eligibility criteria included: (1) being between 18 and 30 years of age and (2) successful clinical completion of two occlusal splints—one fabricated using a digital workflow (involving intraoral scanning and CAD/CAM technology), and the other via a conventional workflow (manual impression‐taking and laboratory processing). Students who declined participation or did not consent to audio recording were excluded.

The total number of students enrolled in the course during the academic term, following a census sampling approach, defined the sample. This strategy ensured that all eligible students were included, thus maximizing representativeness within the defined educational context. Given the qualitative nature of the study—focused on exploring perceptions and learning outcomes—this sample size is methodologically appropriate. Unlike quantitative studies, qualitative research does not require statistical parameters such as effect size, sampling error, or power analysis. Instead, methodological rigor is achieved through thematic saturation, a criterion indicating that no new themes emerge from additional data collection. Prior studies suggest that saturation is typically reached with 12–24 participants in homogeneous samples and narrowly focused inquiries. Therefore, the inclusion of 24 students was considered sufficient to ensure analytic depth and saturation, supporting the study's exploratory goals and contributing to the credibility and trustworthiness of its findings.

### 2.3 Educational Intervention

The intervention required students to complete two occlusal splint fabrication workflows—one digital and one conventional—under faculty supervision. These procedures were incorporated into the practical training module of the course and structured to stimulate comparative analysis, skill development, and reflective practice. By performing both workflows, students were encouraged to assess the practical, clinical, and technical aspects of each method. Reflective discussions were promoted throughout, and the experience was contextualized within the broader learning objectives of prosthodontic education and digital dentistry integration.

In the conventional workflow, occlusal splints were fabricated using the wax‐up and lost‐wax processing technique. The students were responsible for all clinical and pre‐laboratory stages, including impression‐taking, pouring of stone casts, facebow registration, intraoral wax record using a Lucia jig, and mounting the casts on a semi‐adjustable articulator, followed by wax‐up of the splints on the mounted models. The subsequent laboratory procedures—flasking, acrylic resin processing, polymerization, and pressing—were performed by an experienced dental laboratory technician specializing in prosthodontics, with students present to observe these steps. Final finishing of the splints after polymerization was carried out by the technician, while the clinical installation and occlusal adjustments were completed by the students under faculty supervision. This division of responsibilities ensured that students actively engaged in the diagnostic and technical decision‐making phases while also gaining exposure to the laboratory workflow through guided observation.

In the CAD/CAM workflow, intraoral scanning of the maxillary and mandibular arches, as well as the interocclusal registration in maximum intercuspation using a Lucia Jig, was performed by the students with a CEREC scanner (Dentsply Sirona, Brazil, 2022). A standardized scanning protocol was followed to ensure accuracy of the digital model capture, including drying of dental surfaces, separate scanning of the upper and lower arches, and verification of the occlusion scan. The resulting STL files were exported and imported into the Medit Link software (v3.2.2; Medit, South Korea, 2024) for virtual design of the occlusal splints. Automatic design was generated using the software's AI algorithm, after which students carried out manual refinements to adjust parameters such as splint thickness (2–3 mm), full‐arch coverage, and balanced occlusal contacts to optimize stability and functionality.

Once finalized, the designs were transferred to the laboratory for additive manufacturing using the MARS 4 DLP 3D printer (Elegoo, China, 2023) and a biocompatible resin formulated for occlusal splints (Prizma 3D Bio Splint, Marketech Labs, Brazil, 2023). The printing stage was conducted by a trained dental laboratory technician, who also performed post‐processing steps, including washing in 99% isopropyl alcohol, ultraviolet light polymerization in a curing chamber (ciclOne, Done3D, Brazil, 2023), removal of support structures, and preliminary finishing of the printed splints. The students then assumed responsibility for the clinical phases, performing installation and selective occlusal adjustments under faculty supervision. Adjustments were guided by articulating paper markings, ensuring uniform distribution of occlusal contacts in maximum intercuspation as well as appropriate anterior and canine guidance during excursive movements. This division of responsibilities ensured that students actively engaged in all clinical and design stages, while the laboratory technician provided specialized support for high‐precision printing and post‐processing.

### 2.4 Data Collection

#### 2.4.1 Qualitative Data

Immediately following the completion of both fabrication processes, individual semi‐structured interviews were conducted by a faculty member with training in qualitative research. The interviews were guided by two open‐ended questions:

1. “Identify the positive aspects and challenges you experienced while fabricating an occlusal splint using the digital workflow.”
2. “Identify the positive aspects and challenges you experienced while fabricating an occlusal splint using the conventional workflow.”

All interviews were audio‐recorded using the Apple Voice Memos application and lasted approximately 10–15 min. Transcriptions were generated using the ChatGPT‐4.0 platform (OpenAI), then manually reviewed to ensure narrative fidelity and preserve linguistic authenticity. This triangulated transcription process, combining AI‐generated outputs with human verification and software‐based analysis, enhanced the credibility and transparency of the qualitative data.

#### 2.4.2 Quantitative Data

A structured electronic questionnaire was administered via Google Forms immediately after the practical component. Students were asked to rate their experience with each workflow on three dimensions:

1. Ease of fabrication (technical confidence)
2. Satisfaction with the final clinical result (perceived competence)
3. Likelihood of recommendation (professional endorsement)

Responses were recorded on a 0–10 Likert scale, with higher scores indicating more favorable perceptions. This instrument was designed to capture student preferences while reflecting relevant educational competencies in prosthodontic clinical training.

### 2.5 Data Analysis

#### 2.5.1 Qualitative Analysis

Qualitative data were analyzed using IRaMuTeQ software (Interface de R pour les Analyses Multidimensionnelles de Textes et de Questionnaires), which enabled lexical classification, word frequency distributions, and sample dispersion clouds. Content analysis followed Bardin's thematic framework, using five pre‐established educational categories: time and agility, precision and adjustments, patient comfort, cost and accessibility, and learning and skill development. Two researchers independently coded the data, and discrepancies were resolved through discussion to ensure inter‐coder reliability.

#### 2.5.2 Quantitative Analysis

Quantitative data were exported to Google Sheets and analyzed using IBM SPSS Statistics (version 26.0; IBM Corp., Armonk, NY, USA). Descriptive statistics (mean, standard deviation, minimum, maximum, and median) were calculated for each questionnaire item.

Normality of data distribution was assessed using the Shapiro–Wilk test. Depending on the results:

1. Paired Student's t‐tests were used for normally distributed data.
2. Wilcoxon signed‐rank tests were applied for non‐normally distributed data.

Agreement between student evaluations of the two workflows was assessed using Lin's concordance correlation coefficient (CCC) and the intraclass correlation coefficient (ICC) with 95% confidence intervals. Bland–Altman plots were generated to visualize systematic bias and assess agreement across the three dimensions.

## 3 Results

A total of 24 undergraduate dental students participated in the study. Qualitative analysis of their interviews revealed five core educational categories: time and agility, precision and adjustments, patient comfort, cost and accessibility, and learning and skill development.

**FIGURE 1:**
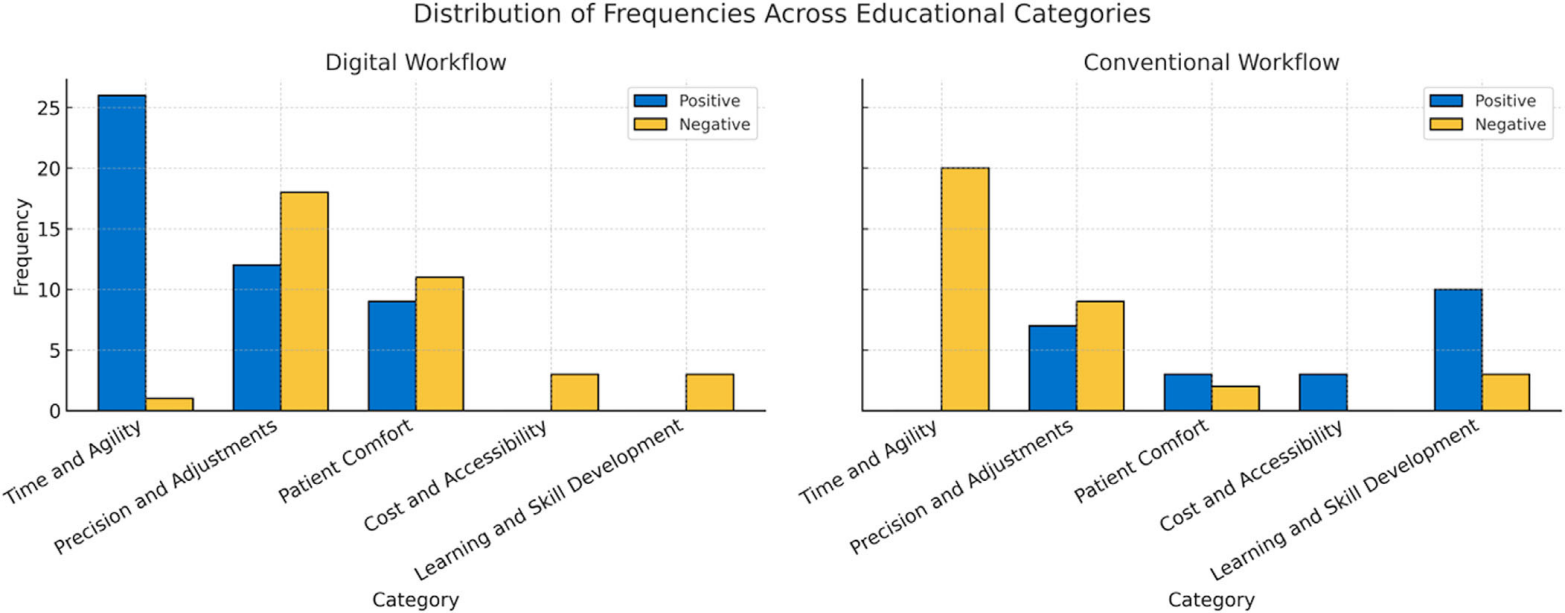
Distribution of student‐reported positive and negative experiences across five educational categories for digital and conventional workflows. The digital method was predominantly associated with gains in procedural efficiency and patient comfort, whereas the conventional method was valued for promoting manual skill development and a deeper understanding of clinical processes.

The distribution of student perceptions across the main educational categories revealed distinct patterns between the digital and conventional workflows (Figure 1). For the digital workflow, positive comments were predominantly concentrated in the domain of time and agility, with 26 favorable citations highlighting the efficiency and reduced chair time associated with digital fabrication. Precision and adjustments, and patient comfort also generated a considerable number of positive mentions, although notable challenges were identified in these domains, particularly concerning intraoral scanning difficulties. In contrast, for the conventional workflow, positive feedback was predominantly observed in the learning and skill development category, with students emphasizing the value of manual experience and procedural understanding gained through traditional fabrication methods. Negative perceptions of the conventional workflow were concentrated on time and agility, with frequent references to the prolonged procedural time and complexity. These patterns underscore that while digital workflows are perceived as more efficient and patient‐friendly, conventional techniques remain highly valued for promoting technical competency and comprehensive clinical learning.

In the domain of *time and agility*, the digital workflow was consistently described as highly advantageous. Students emphasized the reduced chair time, procedural simplicity, and overall agility afforded by digital tools, with twenty‐six positive references to these aspects. Only one participant noted a negative experience related to difficulties during intraoral scanning. In contrast, the conventional method elicited twenty negative comments regarding the extended time required for execution, the greater number of procedural steps, and the dependence on laboratory processes, with no positive aspects mentioned within this category.

Regarding *precision and adjustments*, perceptions were more nuanced. While twelve students highlighted that digital fabrication required fewer occlusal adjustments and provided better initial fit, eighteen participants also pointed out technical challenges, particularly difficulties related to the size of the intraoral scanner, moisture control, and limitations in accessing posterior areas. Conversely, the conventional method received seven positive mentions for providing a higher quality of final finishing and perceived greater material durability, though nine students reported negative experiences, especially involving the need for extensive occlusal adjustments and the complexity of laboratory steps.

In the category of *patient comfort*, the digital workflow was again favorably viewed, with nine students noting advantages such as fewer appointments and the elimination of discomfort associated with traditional impression trays. However, eleven students indicated that the intraoral scanner's size and the need for soft tissue manipulation sometimes caused patient discomfort. For the conventional workflow, references to patient comfort were limited, comprising three positive and two negative citations.

*Cost and accessibility* emerged as limiting factors for the digital workflow, with three students commenting negatively on its cost and limited clinical access. In contrast, the conventional workflow was associated with greater affordability and accessibility, with three positive mentions and no reported negatives.

Regarding *learning and skill development*, the conventional workflow was highly appreciated. Ten students recognized that fabricating occlusal splints manually enhanced their understanding of clinical processes and improved their technical skills. Meanwhile, three students expressed concerns about the challenges of learning digital workflows, particularly the steep learning curve involved in mastering CAD/CAM technology and intraoral scanning procedures.

Overall, the qualitative findings suggest that although students preferred the digital method for its efficiency and patient‐related benefits, they also recognized the conventional workflow as crucial for building foundational clinical skills and understanding the overall complexity of occlusal splint fabrication. These thematic findings are summarized in Table 1, providing an overview of students' experiences with both workflows in each educational category.

**TABLE 1:** Emergent educational themes from qualitative analysis.

Category	Digital workflow	Conventional workflow
Time and agility	Positive: Fast workflow; reduced time	Negative: Long procedure; many steps
Precision and adjustments	Mixed: Less adjustment needed, but scanner challenges	Mixed: Good final finish, but more adjustments
Patient comfort	Positive: No impressions; Fewer appointments; Negative: Discomfort from scanner	Limited discussion; mostly neutral
Cost and accessibility	Negative: High cost; limited access	Positive: More affordable and accessible
Learning and skill development	Negative: Steep learning curve	Positive: Better understanding of procedures and hands‐on skills

*Note*: Categories were established a priori based on educational relevance to occlusal splint fabrication. “Positive” entries indicate students’ favorable experiences with the workflow; “Negative” entries indicate challenges or limitations reported. Analysis was conducted using NVivo software with content coding aligned to Bardin's method for thematic interpretation.

The quantitative analysis supported these findings. When rating their experiences on a 0–10 Likert scale, students reported a mean score of 8.8 (±1.0) for ease of fabrication using the digital method, compared to a mean score of 4.9 (±2.0) for the conventional method. Satisfaction with the final clinical result was high for both methods, with slightly higher scores for the digital workflow (8.9 ± 1.2) compared to the conventional workflow (8.4 ± 1.2). In terms of likelihood of recommendation, the digital method was markedly preferred, with a mean score of 9.6 (±0.9), while the conventional method received a lower score of 6.3 (±3.0), as summarized in Table 2.

**TABLE 2:** Student ratings for digital and conventional workflows.

Dimension	Digital workflow (Mean ± SD)	Conventional workflow (Mean ± SD)
Ease of fabrication	8.8 ± 1.0	4.9 ± 2.0
Satisfaction with the result	8.9 ± 1.2	8.4 ± 1.2
Likelihood of recommendation	9.6 ± 0.9	6.3 ± 3.0

*Note*: Descriptive statistics of students’ evaluations of each fabrication workflow (digital and conventional) across three dimensions: ease of fabrication, satisfaction with the final clinical result, and likelihood of recommendation. Responses were collected using a 0‐to‐10 Likert scale, where higher scores indicate more favorable perceptions. Students reported greater ease and a higher likelihood of recommending the digital method, while satisfaction with the result was similarly high for both workflows.

Notably, the higher standard deviation observed for the conventional workflow, particularly in the likelihood of recommendation (standard deviation = 3.0), suggests greater variability in student perceptions. This dispersion may reflect individual differences in familiarity with manual techniques, confidence in analog procedures, or discomfort with longer chairside times. In contrast, the digital workflow presented more consistent scores across participants, indicating a more uniformly positive experience with digital tools.

However, agreement analyses revealed that students’ evaluations of the two workflows were not strongly concordant. The results of Lin's CCC and ICC are presented in Table 3. Lin's Concordance Correlation Coefficient was low across all items, and the intraclass correlation coefficients indicated moderate consistency, favoring the digital workflow for ease of fabrication, but negative correlations for satisfaction and likelihood of recommendation, suggesting a systematic tendency to favor the digital approach. Bland–Altman plots further illustrated a systematic bias in favor of the digital workflow, particularly for ease of fabrication and recommendation, while satisfaction scores, although slightly favoring the digital method, showed less pronounced differences.

**TABLE 3:** Agreement analysis between digital and conventional workflows.

Dimension	Lin's CCC	ICC (95% CI)	*p*‐value
Ease of fabrication	0.0511	0.340 (−0.526 to 0.714)	0.163
Satisfaction with the result	−0.1588	−0.416 (−2.273 to 0.387)	0.795
Likelihood of recommendation	−0.0803	−0.414 (−2.270 to 0.388)	0.794

*Note*: CCC = Lin's Concordance Correlation Coefficient; ICC = Intraclass Correlation Coefficient; CI = Confidence Interval. Positive ICC values indicate a trend toward agreement between the two methods, while negative values suggest a preference for one method over the other. A p‐value < 0.05 was considered statistically significant. The analysis revealed no significant agreement between digital and conventional workflows across the evaluated dimensions.

The low Lin's CCC and negative ICC values for satisfaction and likelihood of recommendation indicate poor agreement between the two methods. These results suggest that students did not perceive the digital and conventional workflows as interchangeable; instead, they experienced each as a distinct learning process with unique characteristics. Specifically, while ease of fabrication showed moderate consistency favoring the digital method, the marked divergence in satisfaction and recommendation scores points to differing emotional or cognitive responses, reinforcing the idea that each method supports different aspects of the learning experience.

**FIGURE 2:**
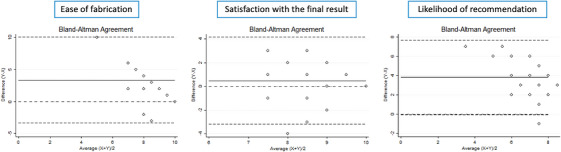
Bland–Altman plots comparing student evaluations of the digital and conventional workflows across three dimensions: ease of fabrication (left), satisfaction with the result (center), and likelihood of recommendation (right). In each plot, the X‐axis represents the average score between the two workflows for each participant (digital+conventional)/2(digital+conventional)/2, while the Y‐axis represents the difference between the scores assigned to the conventional and digital workflows (conventional–digital) (conventional–digital). A systematic bias favoring the digital workflow is observed in both the ease of fabrication and recommendation domains, as indicated by the high mean differences and most points falling below the zero line. In contrast, the satisfaction domain demonstrates closer agreement between methods, with differences clustering around zero. These findings suggest that students perceived the digital workflow as more practical and recommendable, although the perceived clinical outcomes were similarly satisfactory for both approaches.

Figure 2 displays Bland–Altman plots comparing student ratings of the digital and conventional workflows across three domains: ease of fabrication, satisfaction with the result, and likelihood of recommendation. In each plot, the X‐axis represents the average score of the two methods for each participant (digital+conventional)/2(digital+conventional)/2, while the Y‐axis denotes the difference between the methods (conventional–digital) (conventional–digital). A systematic bias favoring the digital workflow was observed in both ease of fabrication and likelihood of recommendation, indicated by positive differences and a consistent deviation from the zero line. This suggests that students clearly preferred the digital method in terms of practicality and endorsement. Conversely, the satisfaction domain showed minimal variation around the mean, indicating that, despite operational preferences, students perceived the clinical outcomes of both methods as similarly satisfactory.

The Bland–Altman plots further illustrate this divergence, with most data points falling below the zero line in the ease and recommendation domains, indicating a systematic bias toward the digital method. This consistent preference may be attributed to the reduced technical demands and shorter procedural time associated with digital workflows. However, the tighter clustering of satisfaction scores around the mean suggests that, regardless of workflow, students felt confident in the final outcomes, possibly due to adequate supervision or curricular design that ensured equivalent end‐product quality.

Lexical content analysis revealed distinct patterns in students’ perceptions across the five pre‐established educational categories, highlighting both the strengths and limitations of each workflow. These thematic distinctions are summarized in Table 4, which organizes student responses according to domains such as time and agility, precision and adjustments, patient comfort, cost and accessibility, and learning and skill development. In the *time and agility* domain, the conventional workflow was predominantly associated with negative terms such as “delay,” “difficulty,” and “takes time,” reflecting students’ frustration with the prolonged duration and procedural complexity of manual techniques. In contrast, the digital workflow elicited positive descriptors like “fast,” “efficient,” and “easy,” indicating a clear perception of streamlined processes and operational advantages.

**TABLE 4:** Representative student quotes illustrating perceptions of digital and conventional workflows across educational themes.

Educational theme	Workflow	Representative quote
Time and agility	Digital	“It was fast and intuitive. I could finish the scan and design in one session.”
	Conventional	“It took too long and had too many steps. I felt like everything depended on the lab.”
Precision and adjustments	Digital	“The splint needed very few adjustments. The initial fit was really accurate.”
	Conventional	“I had to adjust the occlusion a lot; the contacts weren't right after the lab step.”
Patient comfort	Digital	“The scanner was a bit bulky, and it was hard to reach the back molars comfortably.”
	Conventional	“The impression tray was uncomfortable, but it was quicker than I expected.”
Cost and accessibility	Digital	“It's a great tool, but I don't think I'll have access to this kind of technology soon.”
	Conventional	“This is what we'll use more often in real clinics—it's affordable and available.”
Learning and skill development	Digital	“I struggled with the CAD software at first, but I learned a lot about how it works.”
	Conventional	“Doing everything by hand helped me understand each step better—it made the theory real.”

*Note*: Student quotes were selected from transcribed semi‐structured interviews conducted after the completion of both workflows. Each quote was chosen to represent a typical perspective within its respective thematic category, based on content saturation and recurrence across participants. These narratives complement the lexical analysis and support the five pre‐established educational categories used in the qualitative coding framework: time and agility, precision and adjustments, patient comfort, cost and accessibility, and learning and skill development. Minor grammatical corrections were made to preserve clarity while maintaining fidelity to students’ original wording.

In terms of *patient comfort*, students frequently referenced negative aspects of the digital workflow, using terms such as “scanner,” “discomfort,” and “mouth” to describe ergonomic challenges and soft tissue manipulation during intraoral scanning. The conventional method generated fewer mentions in this domain and appeared more neutral overall. When addressing *learning and skill development*, the conventional workflow stood out as pedagogically valuable, with frequent use of terms such as “learn,” “understand,” and “steps,” suggesting its importance in fostering procedural comprehension, manual dexterity, and reflective clinical reasoning. Although the digital workflow was also associated with skill‐building, several students highlighted the learning curve and technical challenges involved. Overall, these lexical and thematic findings underscore the complementary nature of both workflows in prosthodontic education, supporting the integration of digital innovation alongside foundational manual training.

## 4 Discussion

The present study explored undergraduate dental students’ perceptions of conventional and digital workflows in the fabrication of occlusal splints, revealing distinct educational advantages and challenges. The findings indicate a clear preference for the digital workflow, which was perceived as more time‐efficient, practical, and user‐friendly—particularly in terms of ease of fabrication and likelihood of recommendation. However, the conventional workflow was valued for its contribution to learning and skill development, with students highlighting its role in fostering manual dexterity, procedural understanding, and clinical reasoning. Satisfaction was comparable between methods, suggesting students achieved similar perceived quality regardless of technique. Although satisfaction with clinical outcomes did not differ significantly between the two workflows, students expressed greater intent to recommend and adopt digital methods in future clinical practice, suggesting a growing alignment between student expectations and the digital transformation in dentistry. This interpretation is supported by the quantitative analysis, which revealed a clear systematic bias in favor of the digital workflow in both ease of fabrication and likelihood of recommendation. The relatively low concordance between workflows, evidenced by negative ICC values and weak Lin's CCC, suggests that students experienced each method as pedagogically distinct. While the digital method was consistently rated as more practical and recommendable, the conventional method elicited more divergent responses—possibly due to varying levels of comfort, previous exposure, and the cognitive load associated with manual execution.

The observed preference for digital workflows is consistent with contemporary trends in health professions education, where learners increasingly expect technology to enhance interactivity, immediacy, and autonomy [4, 5]. CAD/CAM technology and intraoral scanning not only facilitate procedural efficiency but also offer real‐time visual feedback and promote spatial understanding—elements shown to strengthen learning in complex anatomical domains [3, 6, 7]. These tools are aligned with constructivist learning theories that emphasize learner‐centered environments and active knowledge construction [8].

From an educational perspective, the integration of digital technologies reflects a broader paradigm shift in dental training, moving from instructor‐centered approaches to student‐centered and experiential models. This aligns with calls for curricula that foster autonomy, critical thinking, and adaptability in students as future clinicians [9, 10]. Virtual and haptic simulations, 3D modeling, and immersive digital platforms have shown strong potential in promoting clinical reasoning and psychomotor skills. [11, 12] Furthermore, students’ familiarity with digital environments can increase motivation and reduce cognitive overload, enabling a more effective focus on the development of procedural competence [13, 14].

Previous research has shown that dental students are generally positive towards the integration of digital technologies in the preclinical curriculum, highlighting improvements in motivation and perceived learning outcomes [15, 16]. A successful implementation of a digital dentistry curriculum at a U.S. dental school demonstrates that structured introduction of CAD/CAM and intraoral scanning technologies into the curriculum can enhance student satisfaction and clinical preparedness [17]. The present study expands on these findings by directly comparing students’ experiences with both conventional and digital workflows in a practical setting [15].

At the same time, students’ appreciation of the conventional method reinforces the value of hands‐on experiences in developing tactile awareness, sequencing, and problem‐solving. Consistently, conventional hands‐on training remains essential for developing students’ critical judgment and precision in assessing clinical preparations, a skill not fully replicated by digital tools [18]. These findings align with the literature advocating for experiential and situated learning models in clinical education [9, 14]. The analog approach promotes not only technical competence but also critical reflection, as students navigate procedural uncertainties and material handling. While digital tools may streamline workflows, over‐reliance on automation may reduce opportunities for students to engage in deeper cognitive and psychomotor processing. These findings also underscore that, while digital technologies are well‐received, they do not replace the educational value of traditional workflows. The wider variability in ratings for the conventional method, particularly in the recommendation dimension, may reflect students’ individual learning preferences and prior experiences, but also highlights the pedagogical complexity involved in manual techniques. This reinforces the importance of using quantitative data not only to compare preferences but also to guide curriculum planning that strategically balances efficiency, engagement, and depth of learning.

Word cloud analysis supported these observations by capturing students’ subjective representations of each workflow. Digital workflows were associated with positive terms such as “fast,” “efficient,” and “easy,” reflecting alignment with learners’ desire for simplified processes. In contrast, the conventional method elicited terms related to “learning,” “understanding,” and “experience,” highlighting its value as a tool for foundational training. This duality points to the importance of adopting a blended instructional approach that respects the unique educational contributions of both methodologies [10, 11].

These results carry important curricular implications. Dental programs should avoid the binary choice between digital and conventional instruction and instead focus on pedagogical integration that matches instructional design with learning outcomes. Structured curriculum mapping has been proposed as an effective strategy to ensure alignment between learning objectives and instructional content in dental education [19]. The literature increasingly advocates for hybrid curricula that expose students to evolving technologies while grounding them in fundamental clinical reasoning [2, 3]. Simulation‐based education, supported by realistic clinical contexts, has also been shown to foster improved retention and transfer of knowledge [8, 20]. Simulation‐based education improves knowledge retention and transfer in clinical settings [4].

In addition to the pedagogical insights derived from students’ perceptions, the methodological framework of this study reinforces the credibility and consistency of the findings. The use of a mixed‐methods design, combining quantitative ratings with qualitative content analysis, enabled a multidimensional understanding of learning outcomes and student experiences. The adoption of a census sampling strategy—encompassing all students enrolled in the course—ensured contextual representativeness, while the qualitative component adhered to the principle of thematic saturation, which is typically achieved with 12–24 participants in homogeneous and focused samples. The inclusion of 24 students thus aligns with established qualitative research standards, supporting analytical depth and thematic coherence. Furthermore, data triangulation between Likert‐scale evaluations, lexical analysis, and thematic coding enhanced internal validity by corroborating trends across independent sources. The convergence between quantitative preferences for digital workflows and qualitative reflections on procedural efficiency and skill development reinforces the robustness of the study design. As such, the methodological rigor employed provides a solid foundation for the pedagogical implications proposed and strengthens the translational potential of the results for curriculum innovation in dental education. Moreover, the integration of quantitative metrics such as ICC and Bland–Altman analysis helped uncover subtle pedagogical dynamics that may not be evident through satisfaction scores alone. These analytical tools revealed not only student preferences but also how each workflow elicited unique responses, pointing to their complementary—not redundant—roles in prosthodontic training. Future studies should continue to apply such metrics to triangulate student perception with performance and learning outcomes.

In addition to the limitations already mentioned, it is possible that social desirability bias may have influenced the responses, as students may have felt inclined to favor digital innovations in a highly technological academic environment [21]. First, it was conducted within a single institution and academic cohort, which may limit the generalizability of findings to other educational settings or cultural contexts. Second, although the mixed‐methods design enriched the interpretation of results, the qualitative component relied on self‐reported perceptions, which are subject to bias and influenced by students’ prior experiences with technology. Third, the study did not assess long‐term skill retention or clinical performance, which would be essential for evaluating the sustained impact of each workflow on professional competency. Future research should include multi‐institutional samples, objective performance measures, and longitudinal follow‐up to strengthen the evidence base for educational decision‐making in digital dentistry.

## 5 Conclusions

This study highlights the educational benefits and challenges associated with teaching occlusal splint therapy using both conventional and digital workflows in undergraduate dental education. The results demonstrate that while students favor digital methods for their efficiency and ease of use, they continue to value the pedagogical depth offered by conventional techniques, particularly in developing manual skills and procedural understanding. These findings emphasize the importance of adopting a blended educational model that integrates innovative technologies without compromising foundational clinical training. From a curricular perspective, the incorporation of both analog and digital workflows offers complementary learning experiences that support the development of versatile, reflective, and competent future dentists. By aligning educational strategies with students’ learning preferences and emerging technological standards in dentistry, programs can enhance learner engagement, clinical reasoning, and readiness for modern practice. This study contributes to the growing literature advocating for curricular innovation in dental education. By grounding pedagogical decisions in student feedback and educational theory, institutions can foster more meaningful, adaptable, and effective training environments that meet the evolving demands of 21st‐century dental practice.

## Conflicts of Interest

The authors declare no conflicts of interest.
